# Dual Role of the Tyrosine Kinase Syk in Regulation of Toll-Like Receptor Signaling in Plasmacytoid Dendritic Cells

**DOI:** 10.1371/journal.pone.0156063

**Published:** 2016-06-03

**Authors:** Besma Aouar, Denisa Kovarova, Sebastien Letard, Albert Font-Haro, Jonathan Florentin, Jan Weber, David Durantel, Laurence Chaperot, Joel Plumas, Katerina Trejbalova, Jiri Hejnar, Jacques A. Nunès, Daniel Olive, Patrice Dubreuil, Ivan Hirsch, Ruzena Stranska

**Affiliations:** 1 Centre de Recherche en Cancérologie de Marseille, Inserm U1068, Marseille, France; 2 CNRS, UMR7258, Marseille, France; 3 Institut Paoli-Calmettes, Marseille, France; 4 Aix-Marseille Université, UM105, Marseille, France; 5 Institute of Molecular Genetics, Czech Academy of Sciences, Prague, Czech Republic; 6 Department of Genetics and Microbiology, Faculty of Science, Charles University in Prague, Prague, Czech Republic; 7 AB Science, Paris, France; 8 Institute of Organic Chemistry and Biochemistry, Czech Academy of Sciences, Prague, Czech Republic; 9 Centre de Recherche en Cancérologie de Lyon, Inserm U1052, CNRS UMR5286, Lyon, France; 10 UJF, INSERM U823, University Grenoble Alpes, EFS Rhone-Alpes, Grenoble, France; University Hospital Zurich, SWITZERLAND

## Abstract

Crosslinking of regulatory immunoreceptors (RR), such as BDCA-2 (CD303) or ILT7 (CD85g), of plasmacytoid dendritic cells (pDCs) efficiently suppresses production of type-I interferon (IFN)-α/β and other cytokines in response to Toll-like receptor (TLR) 7/9 ligands. This cytokine-inhibitory pathway is mediated by spleen tyrosine kinase (Syk) associated with the ITAM-containing adapter of RR. Here we demonstrate by pharmacological targeting of Syk that in addition to the negative regulation of TLR7/9 signaling *via* RR, Syk also positively regulates the TLR7/9 pathway in human pDCs. Novel highly specific Syk inhibitor AB8779 suppressed IFN-α, TNF-α and IL-6 production induced by TLR7/9 agonists in primary pDCs and in the pDC cell line GEN2.2. Triggering of TLR9 or RR signaling induced a differential kinetics of phosphorylation at Y352 and Y525/526 of Syk and a differential sensitivity to AB8779. Consistent with the different roles of Syk in TLR7/9 and RR signaling, a concentration of AB8779 insufficient to block TLR7/9 signaling still released the block of IFN-α production triggered *via* the RR pathway, including that induced by hepatitis B and C viruses. Thus, pharmacological targeting of Syk partially restored the main pDC function—IFN-α production. Opposing roles of Syk in TLR7/9 and RR pathways may regulate the innate immune response to weaken inflammation reaction.

## Introduction

Plasmacytoid dendritic cells (pDCs) are a highly specialized subset of dendritic cells that plays a central role at the interface of innate and adaptive immunity. They are important actors in antiviral and antitumor immunity but also potent inducers of autoimmune diseases [1–5]. They express endosomal Toll-like receptor (TLR) 7/9, recognizing ssRNA or CpG containing DNA. TLR signaling leads to secretion of proinflammatory cytokines and chemokines as interleukin (IL)-1, tumor necrosis factor (TNF)-α, IL-6, IL-8, and most importantly type I interferons (IFN)-α/β [6–8].

In addition to TLR7/9, pDC express multiple specific receptors that regulate pDC function and thus prevent aberrant immune responses. These include Fc (FcR) and C-type lectin (CLRs) receptors [9, 10], which signal through the B cell receptor (BCR)-like pathway involving Syk, Mek-Erk1/2, and BLNK [6, 10]. Signaling *via* pDC regulatory receptors (RR) attenuates TLR7/9-induced production of IFN and proinflammatory cytokines [6, 7, 10]. This physiological feedback mechanism of IFN control is hijacked in the pathogenesis of several chronic viral infections and cancers, leading to immune tolerance [7, 11–13]. We have previously shown that interaction of HCV envelope glycoprotein E2 with RR, BDCA-2 (CD303) and DCIR (CLECSF-6) activates B cell receptor (BCR)-like signaling that suppresses TLR7/9-mediated production of IFN-α [13]. We hypothesized that inhibition of BCR-like pathway could restore TLR7/9 signaling in pDCs exposed simultaneously to TLR7/9 and RR agonists [7].

Here, we demonstrate by pharmacological targeting of Syk that in addition to the negative regulation of TLR7/9 signaling *via* RR, Syk also positively regulates TLR7/9 pathway in human pDCs. While ample experimental evidence demonstrate negative effect of RR-mediated BCR-like activation of Syk on TLR7/9 signaling, the mechanism by which Syk acts as a positive regulator of TLR7/9 signaling in pDCs is much less clear. Novel highly specific Syk inhibitor AB8779 suppressed IFN-α and TNF-α production induced by TLR7/9 agonists in primary pDCs and in the pDC cell line GEN2.2 [14]. Triggering of TLR9 or RR signaling induced a differential kinetics of phosphorylation at Y352 and Y525/526 of Syk and a differential sensitivity to AB8779. Consistent with the different roles of Syk in TLR7/9 and RR signaling, a concentration of AB8779 insufficient to block TLR7/9 signaling still released the block of IFN-α production triggered *via* the RR pathway, including that induced by hepatitis B and C viruses. Opposing roles of Syk in TLR7/9 and RR pathways suggest that Syk may fine-tune the innate immune response to weaken inflammation reaction.

## Materials and Methods

### Ethics statement

Peripheral blood mononuclear cells (PBMCs) from healthy anonymous donors were obtained from the Etablissement Français du Sang (EFS). Blood samples were obtained after written consent following the approval of the EFS, Marseille, France and the Centre de Recherche en Cancérologie de Marseille (CRCM) in accordance to the convention signed the 20th May 2014. Human pDC line GEN2.2 was obtained from invaded peripheral blood of one patient as described previously [14].

### Isolation and culture of primary pDCs and pDC line GEN2.2

pDCs from PBMCs of healthy donors were purified and cultured as described previously [15, 16]. Human pDC line GEN2.2 was grown in RPMI 1640 medium supplemented with L-glutamine, 10% FCS, 1% sodium pyruvate and 1% MEM nonessential amino acids, on a monolayer of the murine stromal feeder cell line MS-5 as described previously [14]. For phosphoflow and western blot experiments, GEN2.2 cells were separated from MS-5 feeder cells and serum-starved overnight before stimulation.

### Inhibitors, antibodies and reagents

Syk kinase inhibitor AB8779 was from AB Science (Paris, France). *In vitro*, AB8779 was shown to be as potent as Fostamatinib (R406) with IC_50_ = 0.04 μM (S1 Fig and S1 Table). For *in vitro* pDC stimulation assays CpG-A (ODN 2216), CpG-B (ODN 2006), resiquimod (R848), PMA (all InvivoGen, San Diego, USA), BDCA-2 mAb (Miltenyi Biotech, Paris, France), ILT7 (CD85g) mAb and IgG1 isotype control antibody (eBioscience) were used.

### *In vitro* pDC stimulation

To determine cytokine production, purified primary human pDCs (in the presence of IL-3) or GEN2.2 cells were kept at a concentration of 10^6^ cells/mL aliquoted in 100-μL quantities in 96-well round-bottom culture plates, and stimulated with 4 μg/mL CpG-A, 0.5 μg/mL CpG-B, 0.5 μM R848, 25 ng/mL PMA, 10 μg/mL of BDCA-2 or ILT7 antibody, or with HCV, or HBV overnight. In some experiments, BDCA-2 or ILT7-exposed cells were further crosslinked with goat-anti-mouse F(ab’)_2_ (15 μg/mL) (Jackson ImmunoResearch).

### Production and purification of cell culture-derived HCVcc (JFH-1 3M) and HBV

JFH-1 3M HCVcc particles were prepared and purified as described previously [13, 15]. The HBV particles were concentrated from HBV stably transfected HepG2 cell line, clone 2.2.15 (HepG2.2.15) as described previously [17]. The 8% PEG8000 precipitated HBV supernatant purified by ultracentrifugation through 20, 30, 40, 50% sucrose was resuspended in RPMI 1640 medium to obtain a virus suspension containing 10^12^ HBV RNA copies/mL.

### Determination of Syk phosphorylation by dynamic phosphoflow cytometry

Phosphoflow analysis of cells fixed, permeabilized, and incubated successively with phospho-Syk (Tyr525/526) (C87C1) (Cell Signaling Technology, Danvers, USA) rabbit mAb and anti-rabbit biotinylated antibodies was performed as previously described [13, 16, 18].

### Determination of Syk phosphorylation by immunobloting

Phosphorylation of Syk in the 25 μg cytoplasmic fraction in NP1 lysis buffer (Cell Signaling Technology) was analyzed by Western blotting using monoclonal phospho-Syk (Tyr525/526) (C87C1), or polyclonal phospho-Zap-70 (Tyr319)/Syk (Tyr352) Ab and Syk Ab, all from Cell Signaling Technology. In some experiments, the whole cell lysate was immunoprecipitated with phospho-Tyr mouse mAb (P-Tyr-100, Cell Signaling Technology) according to the manufacturer´s instructions and the immunoprecipitate was analyzed by Western blotting using Syk Ab.

### Determination of secreted IFN-α, TNF-α, and IL-6

The quantities of total IFN-α, TNF-α and IL-6 produced by pDCs or GEN2.2 were measured in cell-free supernatants using human ELISA kits (IFN-α and IL-6 from Mabtech, and TNF-α from BD Biosciences).

### Statistical analysis

Quantitative variables are expressed as the means ± SEM (standard error of the mean). To compare the levels of cytokine production by pDCs, Mann-Whitney two-tailed non-parametric test was used. Two-tailed unpaired Student's *t*-test was used to compare quantitative densitometric analysis of Western blots. Data were analyzed with GraphPad Prism 4 software (GraphPad Software, La Jolla, CA). *p* value ≤0.05 was considered to be significant.

## Results

### Differential activation of Syk by TLR7/9 and RR agonists

**Fig 1:**
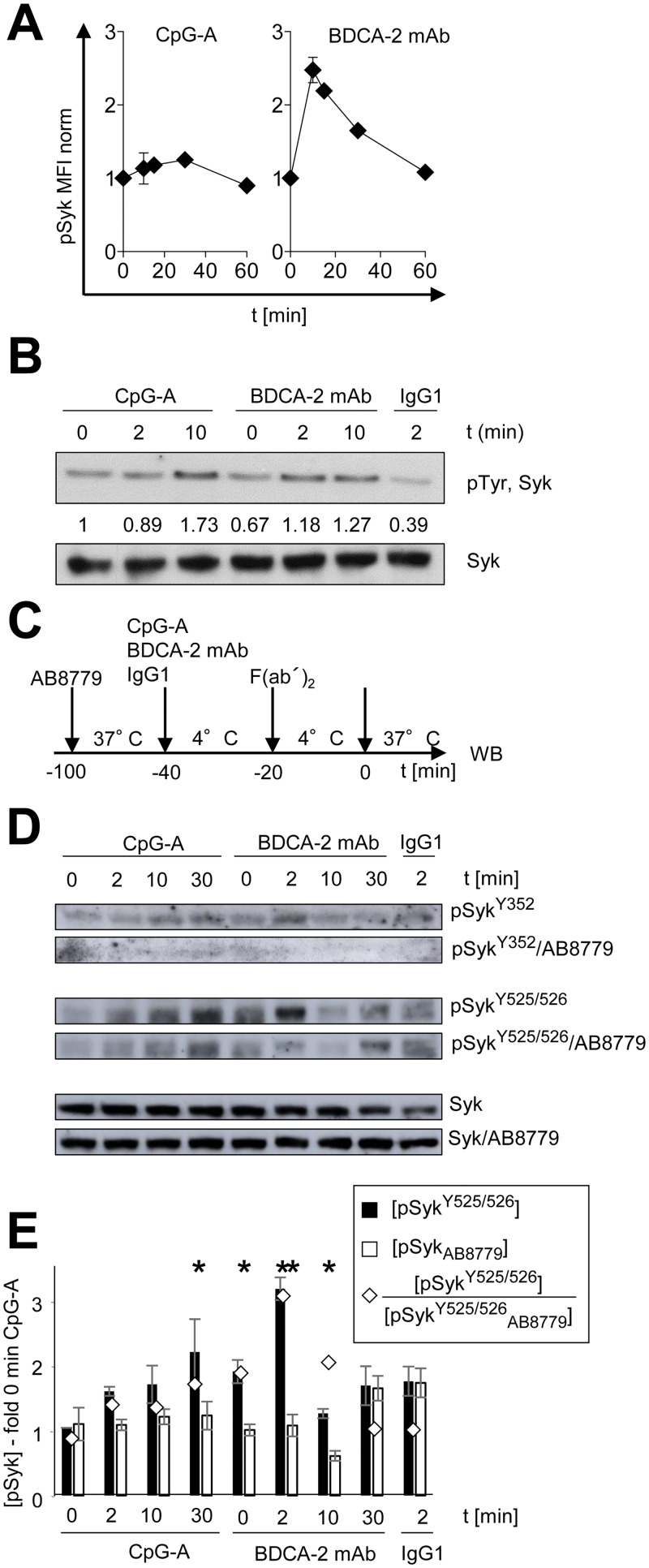
Phosphorylation of Syk in pDCs stimulated by CpG-A or crosslinked with BDCA-2 mAb. (A) Kinetics of phosphorylation of Syk (Y525/526) in the populations of magnetic bead-sorted pDCs exposed to CpG-A or crosslinked with BDCA-2 mAb was followed by flow cytometry (Phosphoflow). The data show means and SEM of three independent experiments with pDCs from different healthy donors. (B) Kinetics of the total Syk phosphorylation in GEN2.2 cells determined by immunoprecipitation of pTyr followed by Western blotting with Syk Ab. Relative quantity of pSyk was determined by densitometry. Total Syk was used as a loading control. (C) Experimental outline. GEN2.2 cells separated from MS-5 feeder cells and serum-starved overnight in RPMI were exposed or not to Syk inhibitor AB8779 for 1.5 h, and then to CpG-A at 4 μg/ml or to BDCA-2 mAb at 10 μg/ml for 20 min at 4°C. BDCA-2-treated cells were crosslinked with F(ab´)_2_ for 20 min at 4°C, and followed by analysis of phosphorylation of Syk by Western blotting. (D) Kinetics of phosphorylation of Syk Y352 (pSyk^Y352^) and Syk Y525/526 (pSyk^Y525/526^) in AB8779-treated or non-treated cells stimulated with CpG-A, BDCA-2 mAb or isotype control (IgG1) was followed by western blot. Total Syk was used as a loading control. Representative result of 3 independent experiments. (E) Quantitative densitometric analysis of phosphorylation of Syk Y525/526 (panel D) in the absence (full columns, [pSyk]) and presence (empty columns, [pSyk_AB8779_]) of AB8779 normalized to the total Syk and expressed as fold increase compared to the control (CpG-A 0 min). ◊, inhibitory index defined by the ratio of pSyk/pSyk_AB8779_ densities. The data show means and SEM, N = 3. *, *p* ≤0.05; **, *p* <0.01; two-tailed unpaired Student's *t*-test.

To investigate the role of Syk in signaling triggered in primary pDCs by TLR7/9 and RR, we compared the kinetics of phosphorylation of Syk induced by agonists of both receptors. As in our previous studies, the insufficient quantities of primary pDCs available for biochemical analyses led us to employ dynamic phosphoflow cytometry [13, 16]. Our results show that triggering of TLR9 and RR signaling differentially induced kinetics of Syk phosphorylation (Fig 1A). TLR9 agonist CpG-A induced phosphorylation of Syk, which gradually increased for 30 min and then decreased to the initial MFI value. In contrast, triggering of RR by crosslinking of BDCA-2 induced rapid phosphorylation of Syk that peaked within 10 min of activation and then decreased during the next 50 min.

To facilitate biochemical analyses of cell signaling, which is still difficult to perform in rare and *in vitro* short living human primary pDCs, we performed our studies in human pDC line GEN2.2, which shares many features with human primary pDCs [14]. First, we compared the kinetics of the total phosphorylation of Syk by immunoprecipitation of the cell lysate with anti-Tyr Ab followed by blotting of immunoprecipitate with Syk mAb (Fig 1B). As in the primary pDCs, triggering of TLR9 and RR signaling in GEN2.2 cells induced differential kinetics of Syk phosphorylation. While after stimulation with CpG-A, total phosphorylation of Syk gradually increased up to 10 min, after stimulation with BDCA-2 mAb, the total phosphorylation of Syk peaked at 2 min.

When cytoplasmic fraction of GEN2.2 cells was probed for phosphorylation of the Syk at Y352 or Y525/526 (Fig 1C), the kinetics of phosphorylation was qualitatively similar to that in primary pDCs and to that of the total Syk phosphorylation in GEN2.2; it gradually increased after stimulation with CpG-A and peaked 2 min after stimulation with BDCA-2 (Fig 1D). While the phosphorylation at Y352, resembled that of the total Syk phosphorylation, the phosphorylation at Y525/526 showed much greater enhancement. In contrast to the total Syk phosphorylation, the phosphorylation of Syk at Y525/526 was more pronounced after stimulation with BDCA-2 than after stimulation with CpG-A, as in primary pDCs (Fig 1A). This result validates GEN2.2 cell line as an appropriate model for the study of cell signaling in pDCs.

The phosphorylation at Y525/526 in the kinase domain of Syk was sensitive to the Syk inhibitor AB8779 (Fig 1D and 1E), which is highly selective compared to Syk inhibitor fostamatinib, R406 (S1 Fig and S1 Table). Densitometric analysis revealed that the inhibitory index defined by the ratio of pSyk^Y525/526^ determined in the absence and the presence of AB8779 ([pSyk^Y525/526^]/[pSyk^525/526^_AB8779_]) reached higher values in BDCA-2 (between 2.1 to 3.0) than in CpG-A-stimulated cells (between 1.5 to 2.1) (Fig 1E). This different effect of inhibitor on the phosphorylation induced by CpG-A and BDCA-2 was not observed for Y352. Taken together, triggering of TLR9 and RR induces differential activation of Syk with a differential sensitivity to AB8779.

### Syk inhibitor blocks TLR7/9-mediated production of IFN-α and proinflammatory cytokines

**Fig 2:**
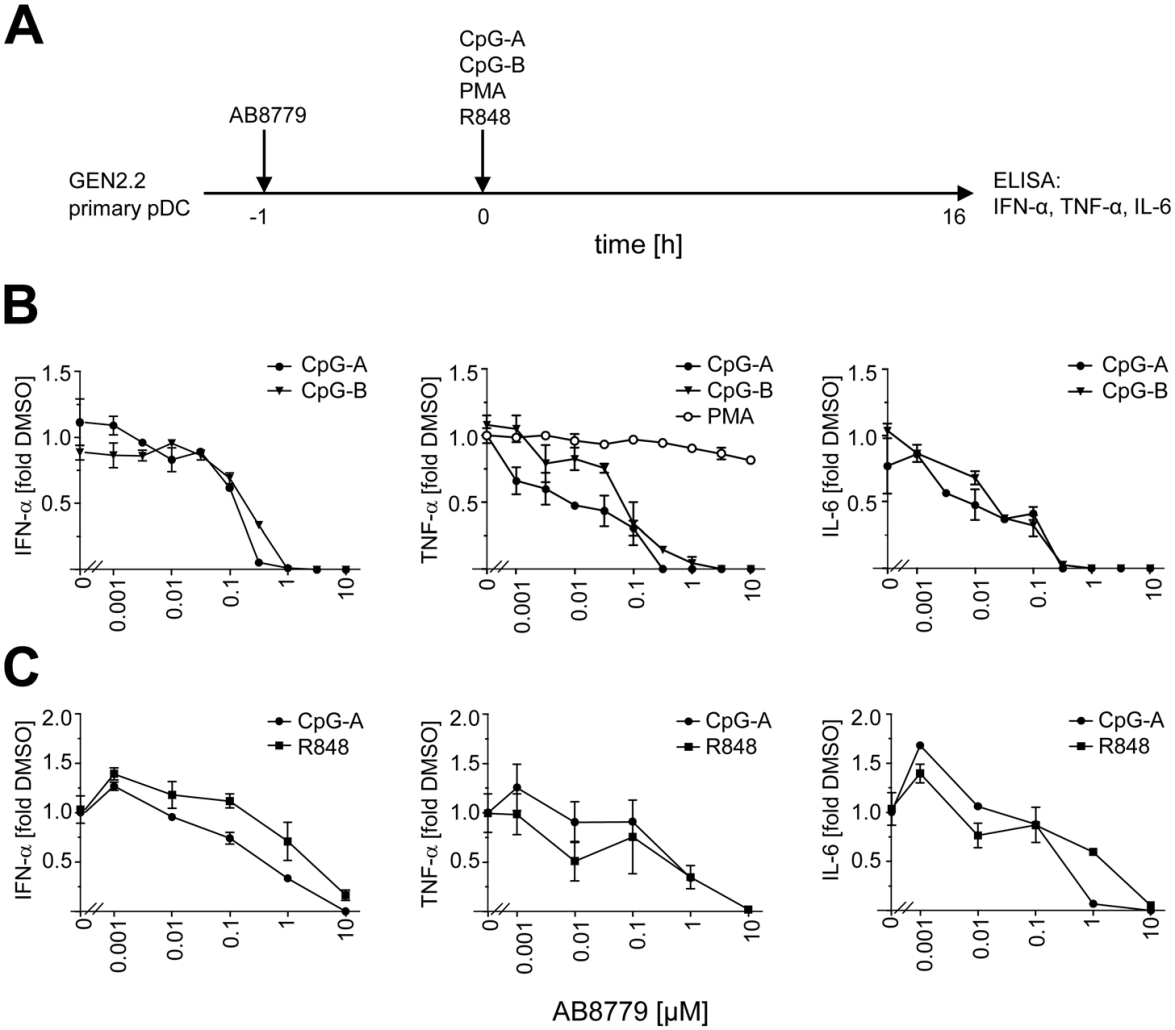
Effect of Syk inhibitor AB8779 on production of IFN-α TNF-α and IL-6 in pDCs. (A) Experimental outline. GEN2.2 cells (B), or primary pDC (C) were incubated with different concentrations of Syk inhibitor AB8779 for 1 hr before stimulation with CpG-A, CpG-B and PMA (N = 3) (B), or CpG-A and R848 (N = 2) (**C**). After 16 hr culture, IFN-α TNF-α and IL-6 production in GEN2.2 cells (B) or in primary pDCs (C) was determined in cell-free supernatants by ELISA and the results expressed as a multiple of control with the matching concentration of DMSO.

We then investigated the effect of AB8779 on cytokine production in GEN2.2 cells stimulated with TLR9 agonists CpG-A and CpG-B, and with protein kinase (PKC) agonist PMA (Fig 2A). AB8779 inhibited IFN-α production with IC_50_,_CpG-A_ = 0.117 μM and IC_50_,_CpG-B_ = 0.215 μM, TNF-α production with IC_50_,_CpG-A_ = 0.006 μM and IC_50_,_CpG-B_ = 0.058 μM and IL-6 production with IC_50,CpG-A_ = 0.023 μM, and IC_50,CpG-B_ = 0.021 μM (Fig 2B). In the same experiments, AB8779 only weakly inhibited production of TNF-α induced by PMA, which does not stimulate IFN-α and IL-6 production. In addition, we investigated effect of AB8779 on cytokine production in primary pDCs from healthy donors. Because synthetic agonists of TLR7 do not induce IFN-α secretion in GEN2.2 cells [14], we used primary pDCs, which permit to assess the effect of AB8779 on both TLR7 and TLR9-mediated cytokine production. As in GEN2.2 cells, AB8779 inhibited production of IFN-α, TNF-α, and IL-6 in primary pDCs stimulated with CpG-A; it inhibited also production of all three cytokines stimulated with synthetic TLR7 agonist R848 (Fig 1C). In sum, Syk inhibition specifically blocked TLR7/9-mediated production of IFN-α and proinflammatory cytokines.

### Subliminal concentrations of Syk inhibitor enhance IFN-α production abrogated by crosslinking of RR by mAbs or by virus particles

**Fig 3:**
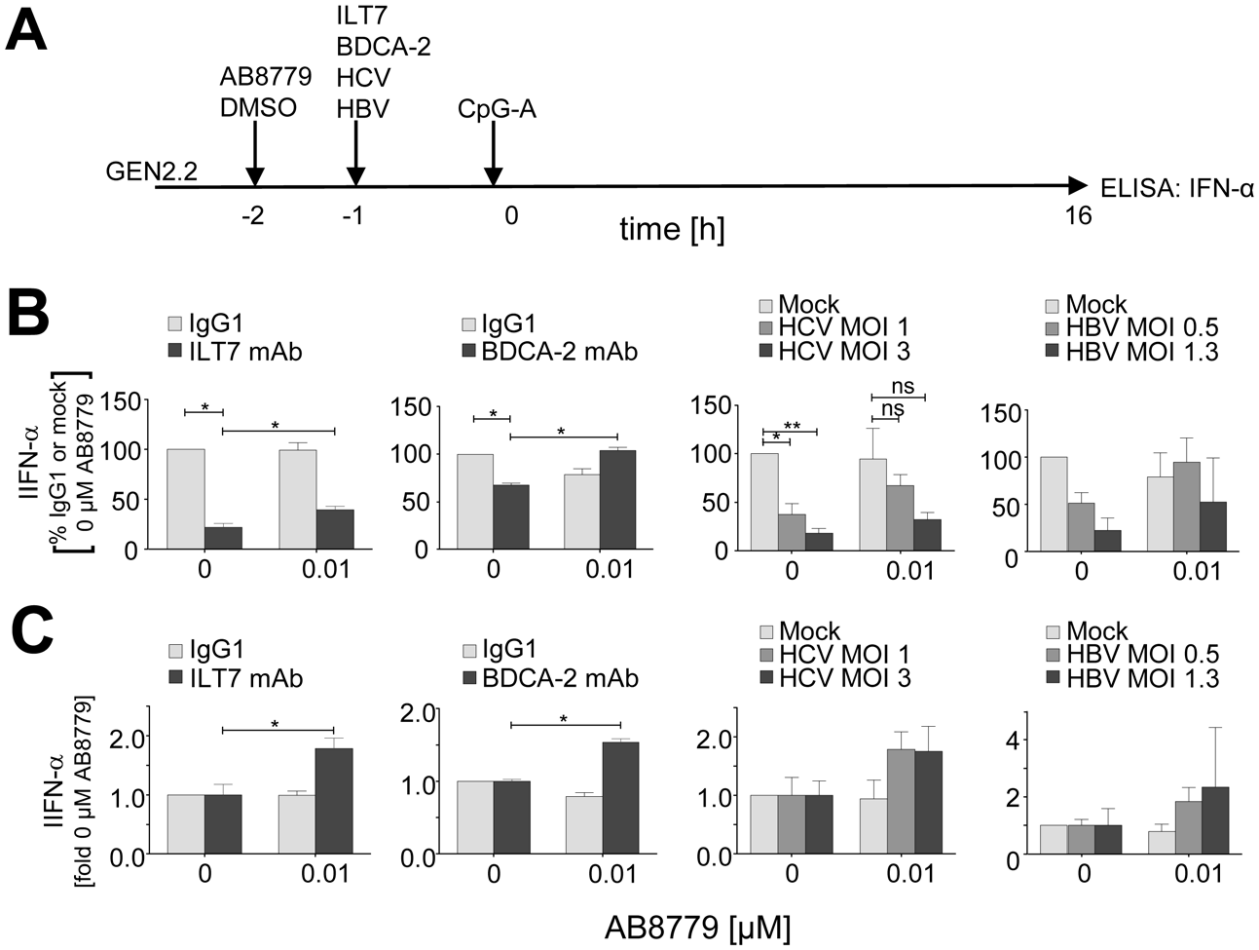
Subliminal concentrations of Syk inhibitor partially restore IFN-α production in GEN2.2 pDC cell line. (A) Experimental outline. After separation from MS-5 feeder cells, GEN2.2 cells were incubated with 0.01 μM AB8779 or with a matching concentration of DMSO for 1 hr before exposure ILT7 or BDCA-2 mAb or HCV or HBV particles and stimulation with CpG-A. (B) After 16 hr culture, IFN-α production was determined in GEN2.2 cell-free supernatants by ELISA, and the results were standardized to the quantity of IFN-α produced by GEN2.2 exposed to isotype control Ab or mock-infected culture in the absence of AB8779 (N = 3). (C) IFN-α production determined in GEN2.2 exposed to ILT7 or BDCA-2 mAb or HCV or HBV particles (shown in B) was normalized to IFN-α production in the absence of AB8779 *, *p* ≤0.05; **, *p* <0.01.

Stronger inhibitory effect of AB8779 on Syk (Y525/526) phosphorylation induced by BDCA-2 than that induced by CpG-A (Fig 1D and 1E) suggested that subliminal concentrations of AB8779, which do not inhibit IFN-α production, could alleviate negative effect of BCR-like signaling on IFN-α production. To test this hypothesis, we exposed GEN2.2 cells pretreated with 0.01 μM AB8779 to ILT7 or BDCA-2 mAbs, or HBV or HCV particles, prior to stimulation with CpG-A (Fig 3A). As expected, in the absence of AB8779, IFN-α production was inhibited by RR crosslinking by mAbs or by HCV/HBV viral particles [12, 13, 15, 19–21] (Fig 3B). After standardization to the quantity of IFN-α produced in the absence of AB8779, pretreatment of GEN2.2 cells with 0.01 μM AB8779 significantly enhanced production of IFN-α (Fig 3C). IFN-α production increased in cells treated with ILT7 (1.8-fold, p = 0.03) and BDCA-2 (1.6-fold, p = 0.04), and it showed a tendency to enhanced production by cells treated with HCV particles (1.8-fold at both MOI = 1 and MOI = 3) and with HBV particles (1.8-fold at MOI = 0.5 and 2.3-fold at MOI = 1.3).

## Discussion

Our results demonstrate that Syk is involved in pDCs in both RR and TLR7/9 signaling. While RR BCR-like pathway represents tolerogenic homeostatic functions of pDCs, TLR7/9 –a pattern recognition receptor pathway is responsible for pDC immunogenic activity. Thus, pharmacological targeting of Syk could be a useful tool to suppress overproduction of IFN-I in autoimmune diseases such as systemic lupus erythematosus and psoriasis, where pDCs play a critical role during initiation of the disease and are an attractive therapeutic target [4, 5].

The role of Syk in TLR signaling has been extensively studied in macrophages/monocytes in the context of the cell membrane localized TLR4 [22–24]. Syk activity is crucial for CD14-dependent endocytosis of TLR4 [22, 23]. Syk-deficient macrophages exhibited decreased TLR4-dependent activation of TBK1 signaling and production of type I IFNs, however, they showed an enhanced activation of TAK1 and increased production of proinflammatory cytokines, compared to that in wild-type macrophages. In contrast, the role of Syk in endosomal TLR7/9 signaling is much less understood. Several reports demonstrated that Syk is recruited to TLR9 upon CpG stimulation, and that this interaction can be blocked by Src kinases inhibitors [23–25]. Because membrane-associated Syk is recruited to ITAM-containing receptors, which is not the case of TLR9, it is likely that the TLR9–Syk association is indirect and that other proteins participate in the formation of a complex. This is compatible with different kinetics of Syk phosphorylation in GEN2.2 cells stimulated with TLR7/9 or RR agonists. Our results show that stimulation of TLR or BCR pathway in pDCs leads to phosphorylation of the activation loop tyrosines Y525/526 that is substantially greater than phosphorylation at Y352 (Fig 1D). A similar observation was made in B cells, where, phosphorylation at Y525/526 was shown to be required for sustained PLC-γ2, Akt and ERK signaling, while phosphorylation at Y352 had a constitutive character [26].

Subliminal concentrations of AB8779, which only weakly inhibited production of IFN-α induced by CpG-A, significantly enhanced production of IFN-α blocked by triggering RR pathway. This suggests that concentration of Syk inhibitor that does not block TLR7/9 pathway (≤0.01 μM AB8779) abrogates negative effect of RR BCR-like signaling on IFN-I production. This result is compatible with different sensitivity of Syk to AB8779 upon stimulation of GEN2.2 cells with TLR7/9 or RR agonists (Fig 1D and 1E); it suggests presence of different pools of Syk in pDCs, one controlling TLR7/9 and the other controlling RR pathway. Restoration of immunogenic activity by pharmacological targeting of Syk is of special interest in the case of pDCs exposed to HBV and HCV particles, where ligation of RR with viruses represents one of the immune escape mechanisms [7, 11–13, 15, 19, 20]. While in the era of the great success of direct acting antivirals against HIV and HCV, stimulation of IFN response might represent an adjuvant therapy, namely important in the case of the virus escape, induction of IFN-I in combination with existing antivirals may cure HBV infection [27].

## Supporting Information

S1 FigTreespot^™^ interaction maps of AB8779 compared to R406 (Fostamatinib).*In vitro* kinase profiling by DiscoverX (Fremont, CA, USA). The result of a high-throughput system (KINOMEscan^™^) for screening of both compounds against large numbers of human kinases (442 kinases) developed by Ambit Biosciences are visualised using a TREEspot^™^ interaction Maps. Kinases found to bind the compounds are marked with red circles, where larger circles indicate higher-affinity binding. The compounds were screened at the concentration of 1 μM, and results for primary screen binding interactions are reported as percent control (% Ctrl), where lower numbers indicate stronger hits. DMSO is used as a negative control (100% Ctrl) while a high affinity compound is used as a positive control (0% Ctrl). % Ctrl is calculated as follow:
$\frac{test\;compound\;signal–positive\;control\;signal}{negative\;control\;signal–positive\;control\;signal}\;\times 100$The S-score of AB8779 tested in this assay is shown in S1 Table. These results clearly show that AB8779 is more specific than Fostamatinib (R406).(PDF)

S1 Table S-score table for AB8779 tested at 1μM. Selectivity (S)-Score is a quantitative measure of compound selectivity. It is calculated by dividing the number of kinases that compounds bind to by the total number of distinct kinases tested, excluding mutant variants. S(35) = (number of non-mutant kinases with % Ctrl <35)/(number of non-mutant kinases tested), S(10) = (number of non-mutant kinases with % Ctrl <10)/(number of non-mutant kinases tested), S(1) = (number of non-mutant kinases with %Ctrl <1)/(number of non-mutant kinases tested).
(PDF)
