# Supplementary material for: Dual Role of the Tyrosine Kinase Syk in Regulation of Toll-Like Receptor Signaling in Plasmacytoid Dendritic Cells
Source: PLoS One. 2016 Jun 3;11(6):e0156063. doi: 10.1371/journal.pone.0156063 (PMC4892542; doi:10.1371/journal.pone.0156063)
Supplement: S1 Fig — In vitro kinase profiling by DiscoverX (Fremont, CA, USA). The result of a high-throughput system (KINOMEscan™) for screening of both compounds against large numbers of human kinases (442 kinases) developed by Ambit Biosciences are visualised using a TREEspot™ interaction Maps. Kinases found to bind the compounds are marked with red circles, where larger circles indicate higher-affinity binding. The compounds were screened at the concentration of 1 μM, and results for primary screen binding interactions are reported as percent control (% Ctrl), where lower numbers indicate stronger hits. DMSO is used as a negative control (100% Ctrl) while a high affinity compound is used as a positive control (0% Ctrl). % Ctrl is calculated as follow: test compound signal–positive control signalnegative control signal–positive control signal ×100 The S-score of AB8779 tested in this assay is shown in S1 Table. These results clearly show that AB8779 is more specific than Fostamatinib (R406). (PDF) [file pone.0156063.s001.pdf]

**R406 @ 1000nM** **AB8779 @ 1000nM**

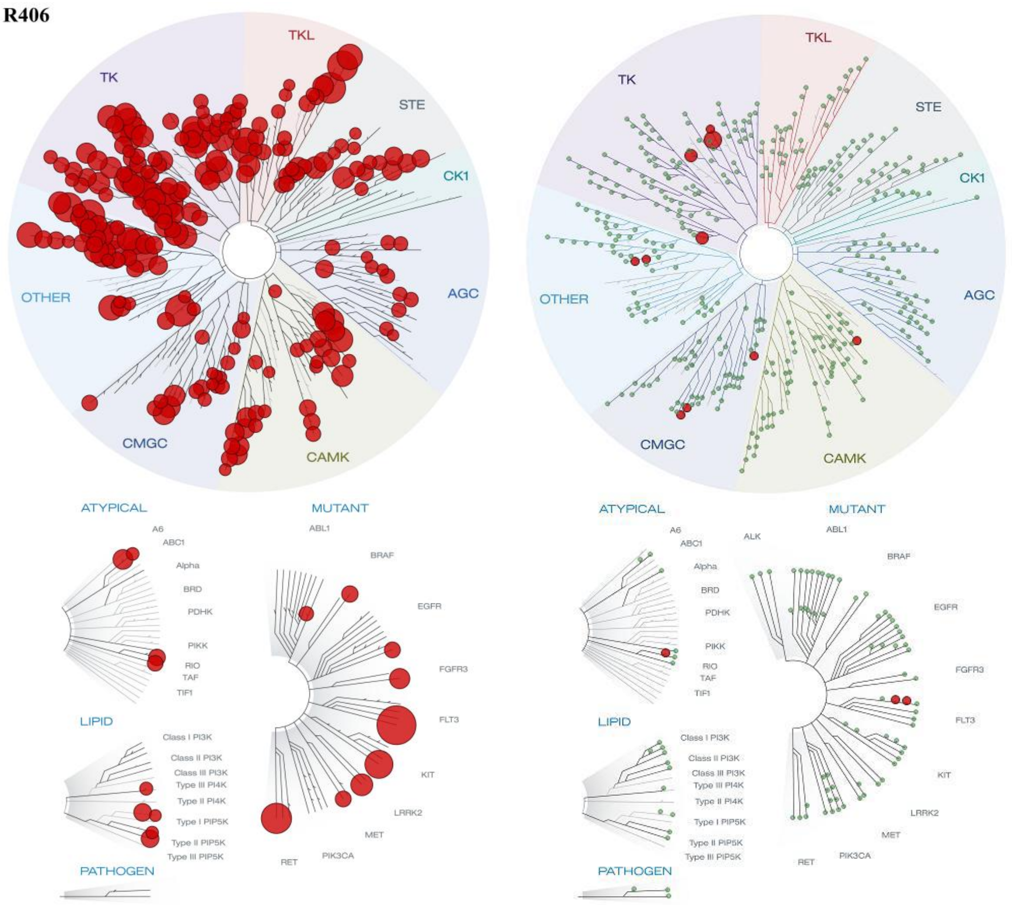


**S1 Fig. Treespot$^{TM}$ interaction maps of AB8779 compared to R406 (Fostamatinib).** *In vitro* kinase profiling by DiscoverX. The result of a high-throughput system (KINOMEscan$^{TM}$) for screening of both compounds against large numbers of human kinases (442 kinases) developed by Ambit Biosciences are visualised using a TREEspot$^{TM}$ interaction Maps. Kinases found to bind the compounds are marked with red circles, where larger circles indicate higher-affinity binding. The compounds were screened at the concentration of 1 µM, and results for primary screen binding interactions are reported as percent control (% Ctrl), where lower numbers indicate stronger hits. DMSO is used as a negative control (100% Ctrl) while a high affinity compound is used as a positive control (0% Ctrl). % Ctrl is calculated as follow:

$$\left(\frac{\text{test compound signal - positive control signal}}{\text{negative control signal - positive control signal}}\right) \times 100$$

The S-score of AB8779 tested in this assay is shown in S1 Table. These results clearly show that AB8779 is more specific than fostamatinib (R406).
