# Supplementary material for: Dual Role of the Tyrosine Kinase Syk in Regulation of Toll-Like Receptor Signaling in Plasmacytoid Dendritic Cells
Source: PLoS One. 2016 Jun 3;11(6):e0156063. doi: 10.1371/journal.pone.0156063 (PMC4892542; doi:10.1371/journal.pone.0156063)
Supplement: S1 Table — Selectivity (S)-Score is a quantitative measure of compound selectivity. It is calculated by dividing the number of kinases that compounds bind to by the total number of distinct kinases tested, excluding mutant variants. S(35) = (number of non-mutant kinases with % Ctrl <35)/(number of non-mutant kinases tested), S(10) = (number of non-mutant kinases with % Ctrl <10)/(number of non-mutant kinases tested), S(1) = (number of non-mutant kinases with %Ctrl <1)/(number of non-mutant kinases tested). (PDF) [file pone.0156063.s002.pdf]

S1 Table. S-score table for AB8779 tested at 1μM

**S-score Type**	**Number of hits/number of non-mutated kinases**	**S-score**
S1	0/395	0
S10	3/395	0.008
S35	11/395	0.028

Selectivity (S)-Score is a quantitative measure of compound selectivity. It is calculated by dividing the number of kinases that compounds bind to by the total number of distinct kinases tested, excluding mutant variants. S(35) = (number of non-mutant kinases with % Ctrl <35)/(number of non-mutant kinases tested), S(10) = (number of non-mutant kinases with % Ctrl <10)/(number of non-mutant kinases tested), S(1) = (number of non-mutant kinases with %Ctrl <1)/(number of non-mutant kinases tested)
